# Community-Based Smoking Cessation Programs: A Way Forward?

**DOI:** 10.3389/fpubh.2019.00364

**Published:** 2019-11-29

**Authors:** Bukunmi Gesinde

**Affiliations:** Department of Health and Behavior Studies, Teacher's College, Columbia University, New York, NY, United States

**Keywords:** vaping, e-cigarettes, public health, smoking, cancer, nicotine, health inequity, health education

In 1950, the association between tobacco smoking and lung cancer risk was first reported (1). Almost 70 years later, it is widely accepted tobacco smoking has a causal relationship for several types of cancer. In 2004, tobacco smoking was officially classified as “carcinogenic to humans” [(1), p. 1]. Cancer develops when the normal processes that regulate human cell behavior are affected by tobacco fail and rogue cells grow uncontrollably. However, there is a considerable discrepancy between awareness and committing to smoking cessation and the actual behavior.

Despite the widespread availability of tobacco cessation public health campaigns, tobacco smoking continues to be one of the main preventable causes of ill-health and premature mortality worldwide. While the general public is aware of the “risks of cigarette smoking, they underestimate” the “addictive nature of cigarette smoking” [(2), p. i14]. Smokers “openly acknowledge the harm they are doing to themselves and many report that they do not enjoy it—yet” they continue to do so [(3), p. 1018]. This occurs because nicotine in cigarettes is a “highly addictive tobacco constituent that is primarily responsible for the maintenance of cigarette smoking” [(2), p. i14].

In July of 2018, the American Public Health Association, Society for Public Health Education (SOPHE), and 38 other public health organizations who recognize tobacco as a preventable cause of illness, actively supported a proposed ruling by the Food and Drug Administration (FDA) to reduce nicotine levels in tobacco products to a minimally addictive or non-addictive level. The FDA and Centers for Disease Control and Prevention's mission to reduce tobacco related death and disease involves public health education campaigns such as Tips From Former Smokers, Fresh Empire Campaign and Every Try Counts Campaign (4, 5). Of note, the Fresh Empire Campaign discourages multicultural youth from tobacco consumption which is often portrayed as a norm in the “hip-hop” demographic.

While noteworthy campaigns have attempted to dissuade smoking, the tobacco industry has a strong influence on low-income communities and cultural norms surrounding smoking. They target communities of color and low-income neighborhoods with heavy marketing and engage in deceptive tactics that allow individuals to remain addicted to smoking. Against this backdrop, we as health educators need to advocate for more stringent regulations on tobacco marketing and retailing practices. We also need to inform the general public how the tobacco industry uses our retail environment to manipulate purchasing behaviors and tobacco consumption. The tobacco industry continues to market their products to minorities, and disproportionately lower-income and socially disadvantaged populations who are already confronted with other stressors of life such as reduced opportunities, racism, microaggressions, and social inequity.

According to Vickers et al. (6), “health disparities are particularly significant across the cancer continuum” with “the burden of cancer in racial and ethnic minorities” from tobacco smoking at historic levels (p. 1). According to Wynn et al. (7), “African Americans continue to bear the disproportionate burden of cancer. Overall, African Americans are more likely to develop cancer and to die from it than any other racial/ethnic” group (p. 55). Furthermore, “African Americans have the highest mortality rate for all cancers combined” (p. 56). “Inequalities in health arise because of inequalities in society—in the conditions in which people are born, grow, live, work, and age” [(8), p. 10]. As public health professionals, we understand health promotion disease results from an accumulation of exposures to risk factors over time. If we as health educators take action during the life course, the systematic inequalities between social groups can be reasonably avoided. According to Marmot (9), “health inequalities that are avoidable and are not avoided are unjust. Putting them right is a matter of social justice” (p. 545).

Community is the individuals, social networks, neighborhoods, local schools, and governmental health providers who work and live together. Community-based programs are an important aspect of public health as they strengthen the health and welfare of committees. These mediating structures are important sources of social resources and social identify and influence a variety of health-related behaviors. They provide an opportunity to integrate evidence-based programs that attempt to work in tandem with communities to improve health equity across various socioeconomic and culturally diverse communities. As health educators, we need to work with communities to promote smoking cessation.

Based on the multilevel framework proposed by McLeroy et al. (10), the five levels of influence specific to health behaviors are intrapersonal factors, interpersonal processes and primary groups, public policy, institutional factors, and of note, community factors. Sustainable health interventions are most successful when community based. Using an ecological model-based framework, social, physical, and cultural factors within a community influence health behavior. Community based health promotion programs use non-traditional settings to educate existing social structures. Additionally, these interventions are able to reach a wide variety of community members without requiring attendance at a traditional medical setting (10). Historically, community-based programs have been used for sexual health education, HIV/AIDS prevention, to increase access to screening and medical management, and to support proper dietary intake. There is no reason why it cannot be successfully applied in the lower-income and socially disadvantaged communities of color to promote an environment of equitable wellness and healthy lifestyle choices for all and to reduce the adverse health outcomes facing our communities and nation.

## Author Contributions

The author confirms being the sole contributor of this work and has approved it for publication.

### Conflict of Interest

The author declares that the research was conducted in the absence of any commercial or financial relationships that could be construed as a potential conflict of interest.
